# Serum Metabolomic and Lipidomic Profiling Reveals the Signature for Postoperative Obesity among Adult-Onset Craniopharyngioma

**DOI:** 10.3390/metabo14060338

**Published:** 2024-06-17

**Authors:** Qiongyue Zhang, Yonghao Feng, Dou Wu, Yinyin Xie, Guoming Wu, Wei Wu, Hui Wang, Xiaoyu Liu, Linling Fan, Boni Xiang, Quanya Sun, Yiming Li, Yongfei Wang, Hongying Ye

**Affiliations:** 1 Department of Endocrinology and Metabolism, Huashan Hospital, Shanghai Medical College, Fudan University, Shanghai 200040, China23211220014@m.fudan.edu.cn (Y.X.); wuwei12@fudan.edu.cn (W.W.);; 2 Department of Endocrinology, Jinshan Hospital, Fudan University, Shanghai 201508, China; 3 Department of Ultrasonography, Shanghai Public Health Clinical Center, Fudan University, Shanghai 201508, China; 4 State Key Laboratory of Genetic Engineering, School of Life Sciences, Fudan University, Shanghai 200433, China; 5 College of Life Sciences, Inner Mongolia University, Hohhot 010021, China; 6 Department of Neurosurgery, Huashan Hospital, Shanghai Medical College, Fudan University, Shanghai 200040, China

**Keywords:** craniopharyngioma, metabolomics, lipidomics, multi-omics, obesity, BMI (body mass index)

## Abstract

Craniopharyngioma patients often suffer from a diminished quality of life after surgery, which is usually associated with metabolic disorders and hypothalamic obesity. However, the precise etiology of these conditions remains elusive. To identify the metabolic changes after surgery, we conducted a cross-sectional study using metabolomic and lipidomic analysis to profile metabolic alterations in adult-onset craniopharyngioma patients with postoperative obesity. A cohort of 120 craniopharyngioma patients who had undergone surgery were examined. Differential analyses, including clinical characteristics, serum metabolome, and lipidome, were conducted across distinct body mass index (BMI) groups. Our findings indicated no statistically significant differences in age, sex, and fasting blood glucose among postoperative craniopharyngioma patients when stratified by BMI. However, a noteworthy difference was observed in uric acid and blood lipid levels. Further investigation revealed that alterations in metabolites and lipids were evidently correlated with increased BMI, indicating that postoperative obesity of craniopharyngioma patients affected their whole-body metabolism. Additionally, the multi-omics analysis identified specific metabolites and lipids, including uric acid and DG(18:2/20:4), as contributors to the metabolic disorders associated with postoperative obesity of craniopharyngioma patients. This work provides valuable insight into the involvement of metabolites and lipids in metabolic disorders subsequent to craniopharyngioma surgery.

## 1. Introduction

Craniopharyngioma is an intracranial tumor that is the leading cause of hypothalamic obesity (HO) and is often accompanied by disorders in glucose and lipid metabolism [1]. The main clinical manifestations of craniopharyngioma are endocrine dysfunction and visual impairment caused by increased intracranial pressure and tumor space. The imaging features of craniopharyngioma are variable, often with cystic degeneration or calcification. A definitive diagnosis requires surgical pathology. Craniopharyngioma accounts for 1.2–4.6% of all brain tumors, yet they exhibit a notably higher recurrence rate compared to pituitary tumors [2,3]. At present, surgery is the principal treatment, supplemented by radiotherapy. Advancements in technologies like nasal endoscopic technology and multidisciplinary team approaches have increased the success rate of surgeries and the survival rate of craniopharyngioma. However, it has been found that the quality of life of patients after craniopharyngioma surgery is low [4]. Our previous study indicates an increased prevalence of metabolic syndrome such as hyperlipidemia, diabetes, and obesity, which greatly affected patient survival and quality of life [5]. Notably, hypothalamic damage can cause abnormal weight gain, known as HO [6], and about 30–70% of craniopharyngioma patients develop HO after surgery [7]. No relevant biomarkers have been reported to predict treatment response and prognosis directly. Currently, diagnosing HO and metabolic disorders requires long-term and rigorous post-operative reexamination of body weight, blood glucose, lipids, and comprehensive examination. However, the curative effect is minimal. There is an urgent need to explore the underlying reasons and specific mechanisms behind the occurrence of metabolic disorders, including HO, after craniopharyngioma surgery.

It has been reported that hypothalamus and pituitary damage, pituitary hormone deficiency, and lifestyle changes may contribute to postoperative obesity in craniopharyngioma patients [8,9,10]. However, small clinical studies using weight loss drugs and bariatric surgery have failed to yield long-term, safe, and satisfactory results [7,11,12]. Moreover, obesity and impaired lipid metabolism in this population are associated with an elevated risk of cardiovascular disease, resulting in higher postoperative mortality rates in craniopharyngioma patients compared to the general population [13]. An improved understanding of lipid and other metabolic abnormalities in craniopharyngioma patients is crucial for uncovering the origins of obesity.

Metabolomics and lipidomics [14,15], as emerging omics research techniques, enable sensitive monitoring and in-depth analysis of small molecular substances in organisms closely related to phenotypes through high-sensitivity, high-resolution, and high-throughput mass spectrometry [16]. Multi-omics technology is increasingly essential in revealing the disease pathogenesis, assessing treatment efficacy, and predicting patient prognosis [17,18]. Mass spectrometry monitoring and analysis have seen rapid advancements [19]. Therefore, utilizing advanced multi-omics technology to analyze the metabolic profile of patients after craniopharyngioma surgery can help reveal the molecular mechanism of HO and establish effective prevention and treatment measures.

This study, primarily through the combined analysis of multiple omics [20], offers more significant insights into the differences in patients’ metabolic profiles after craniopharyngioma surgery. It also lays the groundwork for predicting the postoperative quality of life and devising improvement strategies.

This study aims to investigate the metabolic phenotypes in postoperative craniopharyngioma patients, monitoring serum metabolomics and lipidomic variations. This approach seeks to uncover changes in metabolites and lipids among patients with different metabolic phenotypes to identify biomarkers. This may guide in predicting postoperative weight and preventing metabolic disorders in postoperative craniopharyngioma patients.

## 2. Materials and Methods

### 2.1. Study Populations and Workflow

**Figure 1:**
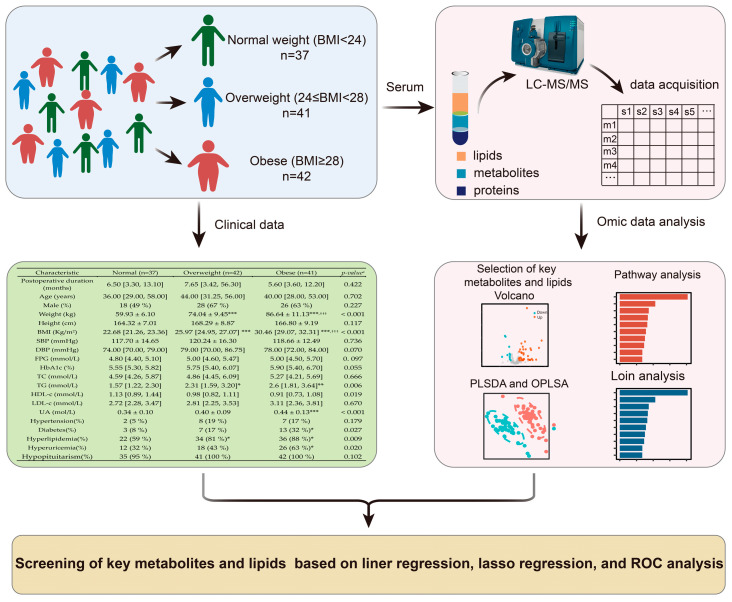
Outline of study workflows. *p*-value ^a^ refer to comparison among three group; * *p* < 0.05, ** *p* < 0.01, *** *p* < 0.001 vs. normal group with Bonferroni correction; ††† *p* < 0.001 vs. overweight group with Bonferroni correction.

A cross-sectional study was conducted at the Department of Endocrinology, Huashan Hospital, Fudan University. Serum samples of 120 postoperative Chinese craniopharyngioma patients were analyzed. Given that Chinese individuals tend to exhibit elevated levels of body fat and higher prevalence of cardiovascular risk factors and all-cause mortality in comparison to white individuals at given body mass index (BMI) levels, lower BMI thresholds of 24 kg/m^2^ and 28 kg/m^2^ are employed as criteria for defining overweight and obesity among the adult population in China [21]. Therefore, serum metabolomics and lipidomics were compared across three BMI categories: <24, 24 to 28, and ≥28. The variances in metabolites and lipids were analyzed, and their correlation with clinical phenotypes was examined. Pregnant women were excluded. The workflow of the study is shown in Figure 1. The Ethics Committee approved the study design of Huashan Hospital at Fudan University (No. 2020727). All of the subjects voluntarily participated in the research and signed informed consent.

### 2.2. Medical Assessment and Blood Sample Collection

Demographics and clinical data, including age, gender, BMI, medical history, medication utilization, diagnosis, and biochemical indicators of glucose and lipid metabolism, were obtained from all subjects (Table 1 and Table S1). The blood samples were drawn in a fasting and resting state, and the fast blood glucose levels were measured using an automatic biochemical analyzer (Hitachi 7600, Tokyo, Japan). The other medical assessments, such as systolic blood pressure and diastolic blood pressure, were performed on the three groups.

### 2.3. Serum Metabolites and Lipids Extraction and LC-MS/MS Analyses

Serum samples were thawed on ice after being stored at −80 °C until the extraction of metabolites and lipids. The protocol using the three-phase system with methyl tert-butyl ether (MTBE) was employed for metabolites and lipid extraction from serum samples using the following steps [22,23]. Step 1: Serum samples (50 μL) were introduced into a 15 mL glass tube containing 0.5 mL high-performance liquid chromatography (HPLC)-grade MTBE. After the addition of (i) 350 μL HPLC-grade water, (ii) 1.5 mL 80% methanol pre-cooled at −80 °C, and (iii) 5 mL HPLC-grade MTBE, tubes were vortexed for 1 min at room temperature and mixed on a rotational table for one hour. Step 2: 1.25 mL HPLC-grade water was added to a separate 15 mL glass tube. After vortexing for 1 min at room temperature, the mixture underwent a 10-min, 1000× *g* centrifugation at 4 °C. Step 3: The top layer organic phases were collected for lipids extraction, and the middle polar layer was collected for metabolomics analysis. Then, a nitrogen-blowing instrument was used to dry the phases overnight, followed by storage at −80 °C.

The metabolite samples were analyzed by liquid chromatography combined with tandem mass spectrometry (LC-MS/MS) on AB SCIEX QTRAP 5500+ (Sciex™, Danville, VA, USA) mass spectrometer coupled to an AB SCIEX ExionLC^TM^ AD LC (Sciex™, Danville, VA, USA) system using a polarity switching approach, which referred from published multiple reaction monitoring list containing 297 transitions [20,24]. The Waters XBridge Amide (Waters Corporation, Milford, CT, USA) (4.6 × 100 mm^2^ i.d., 3.5 μm) was used for LC separation. Mobile phase A was 5% acetonitrile (containing 20 mM ammonium acetate and 0.2% ammonia), and mobile phase B was 100% acetonitrile. 5 μL sample was injected and separated with a 25 min gradient. The column flow rate was maintained at 400 μL/min with a column temperature of 40 °C. The electrospray ionization mass spectra were acquired in positive and negative ion modes, respectively. The multiple reaction monitoring acquisition methods were used to collect MS information simultaneously. The ion spray voltage was set to 4850 V for positive mode, and 4500 V for negative mode, and the heated capillary temperature was maintained at 475 °C. The curtain gas flow, nebulizer, and heater gas were set to 25, 33, and 33 arbitrary units, respectively. All MS files were processed using MultiQuant 3.0.3 (Sciex™, Danville, VA, USA) for peak detection, alignment, and normalization.

Lipid samples were reconstituted using 200 µL of 2-propanol:acetonitrile: water (v:v:v 30:65:5). Then 5 µL of reconstituted sample was injected into the LC-MS/MS. The untargeted lipidomics method was modified from a published method that used a C30 column (Acclaim C30, 3 µm, 2.1 × 150 mm^2^) (Thermo Fisher Scientific, Sunnyvale, CA, USA) [25]. The column flow rate was maintained at 260 μL/min with a column temperature of 45 °C. The LC method used two elution solutions: Mobile phase A (40% water and 60% acetonitrile with 0.1% formic acid and 10 mM ammonium formate) and Mobile phase B (10% acetonitrile and 90% 2-propanol with 0.1% formic acid and 10 mM ammonium formate). The LC gradient with a flow rate of 260 μL/min was initiated as follows: 0 to 1.5 min, 32% solvent B; 4 min, 45% B; 5 min, 52% B; 8 min, 58% B; 11 min, 66% B; 14 min, 70% B; 18 min, 75% B; 21–25 min, 97% B; 25–32 min, 32% B. The sample analysis was performed using an Orbitrap Exploris 480 (Thermo Fisher Scientific, San Jose, CA, USA) mass spectrometer, employing a polarity switching approach with data-dependent acquisition mode. Subsequently, all lipidomics. Raw files were processed using LipidSearch 4.0 (Mitsui Knowledge Industry, Tokyo, Japan) for lipid identification.

### 2.4. Metabolite Pathway and Lipid Ontology Enrichment

The Kyoto Encyclopedia of Genes and Genomes (KEGG) database (http://www.genome.jp/kegg/, accessed on 1 November 2023) was utilized for KEGG pathway enrichment analysis to identify highly enriched metabolic pathways among differential metabolites. Lipid ontology enrichment analysis was performed using the Lipid Ontology (http://lipidontology.com/, accessed on 14 April 2023) to identify highly enriched lipidomic ontology terms among differential lipids. Pathways or lipid ontology terms with a *p*-value < 0.05 were considered significantly altered and were subjected to further analysis.

### 2.5. Statistical Analysis

R software (https://www.r-project.org/, accessed on 31 October 2023, version 4.3.2) was used to analyze the differences in clinical data and omics data among groups. The Kolmogorov-Smimov test was performed first. Continuous variables of the normal distribution were described by Means ± standard deviation (SD). Student’s *t*-test was used to compare the differences between the two groups, and a one-way analysis of variance was used to compare the differences among multiple groups. Non-normally distributed data were represented as medians (interquartile intervals). Wilcoxon Rank Sum Tests were used to compare the differences between the two groups, and the Kruskal-Wallis test was used to compare the differences among multiple groups. Categorical variables were compared using the chi-square test.

Metabolites and lipids with over 70% missing ratios in a particular patient group were removed. Missing values were imputed with one-fifth of the minimum value. Log_2_ fold-change (log_2_FC) was calculated on the mean of the same patient group for each pair of comparing groups. The metabolomic and lipidomic data was log-transformed and autoscaled to ensure proper normalization and to mitigate the impact of variable scale differences using the R package MetaboAnalystR (version 4.0). The partial least squares discriminant analysis (PLSDA) and orthogonal projection to latent structure discriminant analysis (OPLSDA) model were applied using the R package MetaboAnalystR (version 4.0). The model verification was performed by a permutation test repeated 1000 times. In general, *p*-value < 0.05 indicated the available model. Differential metabolites and lipids were identified based on variable importance in projection (VIP), using a score cutoff of >1. Students’ *t*-tests and fold changes were also applied to measure the significance of each metabolite and lipid. Statistical significance was analyzed using a one-tailed Student’s *t*-test, adjusted *p*-values were calculated using the Bonferroni method, and |log_2_FC| > 0.25 and adjusted *p*-value < 0.05 was statistically significant.

The correlation between differential metabolites or lipids and clinical characteristics was assessed using Spearman correlation analysis. Multiple linear regression models were constructed using the Best Subset method to select non-collinear variables, while a minor absolute shrinkage and selection operator (LASSO) regression was employed for the identification of collinear variables. Receiver operating characteristic (ROC) analysis was conducted to predict the occurrence of postoperative obesity in craniopharyngioma patients, and ROC curves were utilized to assess biomarker performance. All regression and ROC analyses were performed with adjustments for age, gender, postoperative time to serum collection, drug treatment, comorbidities, and differential characteristics.

## 3. Results

### 3.1. Study Design and Participants

The study cohort comprised 120 patients who underwent craniopharyngioma surgery, encompassing 37 individuals with normal weight, 41 with overweight status, and 42 classified as obese. A comprehensive summary of pertinent patient demographics is presented in Table 1 and Table S1. The primary outcome revealed no statistically significant differences in postoperative times, age, height, systolic blood pressure, diastolic blood pressure, or gender among the three patient groups. However, these groups’ BMI or weight was statistically significant (adjust *p* < 0.001). Regarding glucose and lipid metabolism, discernible distinctions were observed solely in high-density lipoprotein cholesterol (HDL-c) and triglyceride (TG) levels among the three patient groups. Conversely, fasting blood glucose, glycated hemoglobin, and low-density lipoprotein cholesterol demonstrated no statistically significant differences. Furthermore, there are differences in uric acid (UA) levels among the three patient groups. Given that drug treatment and other disease states can significantly impact metabolic parameters, we conducted a detailed statistical analysis of the three group participants’ medication profiles and disease conditions. The results indicated no significant statistical differences in medication usage or prevalence of other diseases among the three patient groups (Table S1). This suggests that medication usage or other diseases have no significant influence on the metabolic parameters of this study. The serum samples from all group participants were collected for extracting lipids and metabolites and measured using the LC-MS/MS system. T-tests, OPLS-DA, and PLS-DA analyses were conducted to differentiate the metabolomic and lipidomic profiles among participants with different BMIs. Subsequently, correlation analysis and machine learning algorithms were employed to identify metabolites and lipids closely associated with BMI, TG, HDL-c, and UA (Figure 1).

### 3.2. Serum Metabolomic Alterations with Increasing BMI of Postoperative Craniopharyngioma Patients

**Figure 2:**
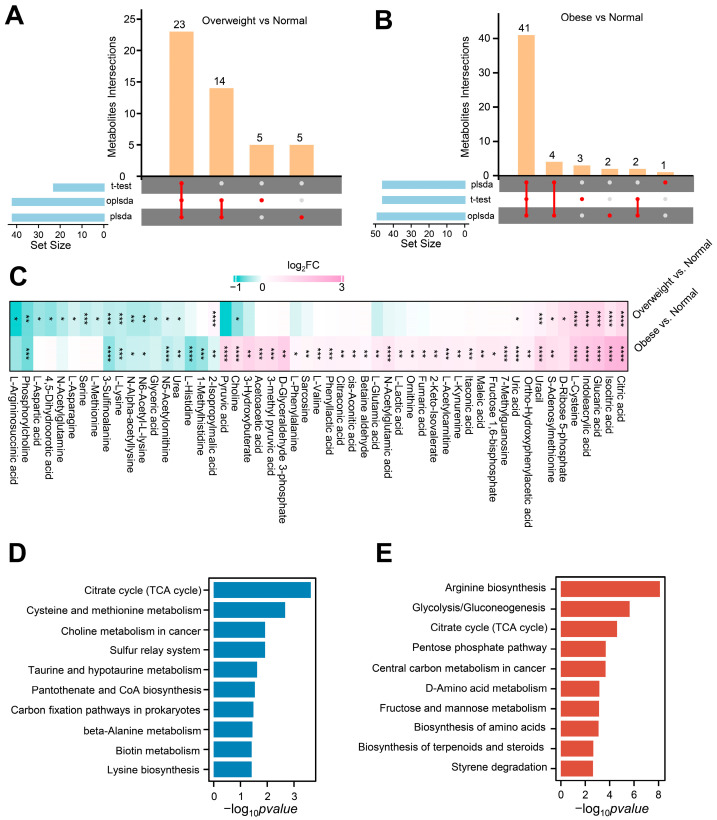
Serum metabolomic alterations with increasing BMI of postoperative craniopharyngioma patients. (**A**,**B**) Identification of differential metabolites between overweight (**A**) or obese (**B**) and normal group by using three distinct statistical algorithms including *t*-test, PLSDA, and OPLSDA. (**C**) Heatmap plots common and distinct differential metabolites in the overweight and obese group compared to the normal group. Each row represents comparisons between different groups, and each column corresponds to another metabolite. The color scale indicates the values of log_2_FC, with red representing higher values and green representing lower values. * adjust *p* < 0.05, ** adjust *p* < 0.01, *** adjust *p* < 0.001, **** adjust *p* < 0.0001. (**D**) Enriched KEGG pathways of differential metabolites shared in both comparisons of obese vs. normal and overweight vs. normal. (**E**) Enriched KEGG pathways of the unique differential metabolites in the comparison of obese vs. normal.

For participants with different BMIs, we analyzed the metabolites that underwent a significant change with the *t*-test, PLSDA, and OPLSDA methods. A total of 148 metabolites from three groups were identified and classified into 14 subcategories (Figure S1A). For overweight vs. normal, 23 of the total 148 metabolites were significantly different, as determined by the *t*-test (Figure 2A and Figure S1B, and Table S2). The PLS-DA and OPLS-DA results show distinct clustering of overweight and normal-weight participants (Figure S1C,D). Using a VIP threshold greater than 1, a total of 42 key metabolites were identified by PLS-DA and OPLS-DA, respectively (Table S2). Then, we made an overlap for differential metabolites derived from the three methodologies, yielding 23 discriminative metabolites, with nine upregulated and 14 downregulated (Figure 2A and Table S2). Subsequently, we conducted the same analysis for obese vs. normal. The *t*-test determined 46 differential metabolites from the total 148 metabolites (Figure 2B and Figure S1E, and Table S3). Meanwhile, the PLS-DA or OPLS-DA filtered 46 or 49 key metabolites using the VIP approach with a threshold greater than 1 (Figure S1F,G, and Table S3). Overlapping analysis for differential metabolites derived from the three methodologies, a total of 41 key metabolites were identified, with 34 upregulated and seven downregulated (Figure 2B and Table S3). We found a concomitant rise in the number of differential metabolites with increasing BMI, primarily characterized by an increase in upregulated metabolites (Figure 2C). It has been shown that certain metabolites are strongly associated with weight gain in postoperative craniopharyngioma patients.

### 3.3. Metabolome KEGG Enrichment Analysis of Serum from Postoperative Craniopharyngioma Patients

For an in-depth analysis of the metabolomic data, all discriminative metabolites identified from the comparisons of obese vs. normal and overweight vs. normal were categorized into those shared by both comparisons and those unique to the comparison of obese vs. normal (Figure S1H). We performed the KEGG pathway analysis to annotate further the potential function of these shared and unique differential metabolites. The differential metabolites commonly observed in both comparisons were enriched in 10 metabolic pathways, and the most critical three pathways were highlighted, including citrate cycle, cysteine and methionine metabolism, and choline metabolism in cancer (Figure 2D). This suggests a significant change in energy synthesis in postoperative craniopharyngioma individuals undergoing weight gain. Meanwhile, cysteine, methionine, and choline metabolism also critically contribute to the weight change in postoperative craniopharyngioma patients. Compared to the overweight vs. normal weight comparison group, the unique differential metabolites in the comparison of obese vs. normal were significantly enriched in another 10 pathways focusing on three pathways: arginine biosynthesis, glycolysis/gluconeogenesis, and citrate cycle pathway (Figure 2E). This implies that a more pronounced increase in body weight in postoperative craniopharyngioma patients significantly affected the arginine biosynthesis, glycolysis/gluconeogenesis, and citrate cycle.

### 3.4. Serum Lipidomic Changes with Increasing BMI of Postoperative Craniopharyngioma Patients

**Figure 3:**
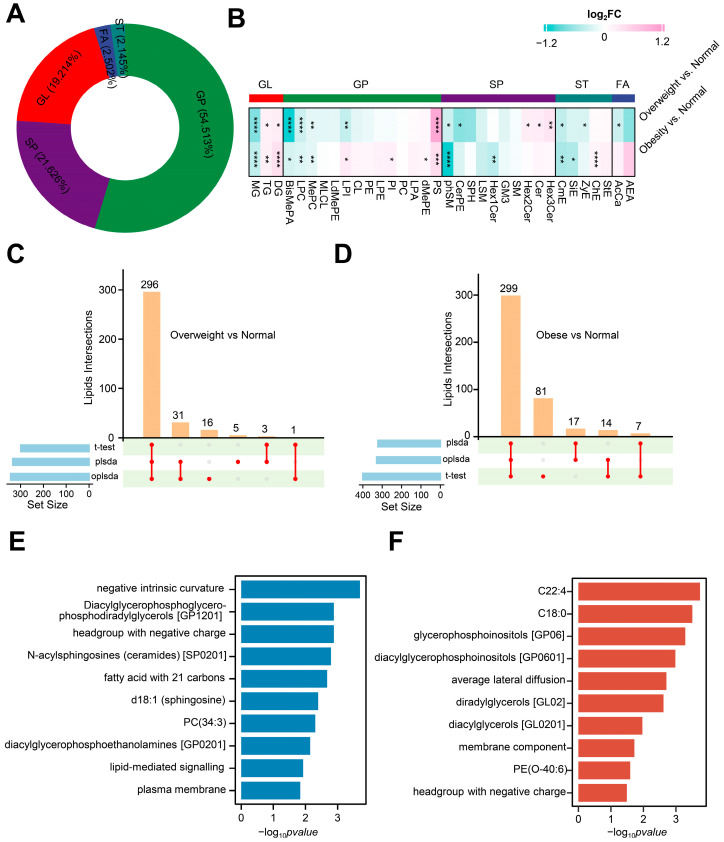
Serum lipidomic changes with increasing BMI of postoperative craniopharyngioma patients. (**A**) Pie charts illustrating the percent of each lipid superclass. GL, glycerolipids; GP, glycerophospholipids; SP, sphingolipids; ST, sterol lipids; FA, fatty acyls. (**B**) Heatmap plots differential lipids in the overweight and obese groups compared with the normal group. Each row represents comparisons between different groups, and each column corresponds to a lipid subcategory. The color scale indicates the values of log_2_foldchange, with red representing higher values and green representing lower values. * adjust *p* < 0.05, ** adjust *p* < 0.01, *** adjust *p* < 0.001, **** adjust *p* < 0.0001. MG, monoacylglycerol; TG, triacylglycerol; DG, diacylglycerol; BisMePA, bis-methyl phosphatidic acid; LPC, lysophosphatidylcholine; MePC, methylphosphatidylcholine; MLCL, monolysocardiolipin; LdMePE, lysodimethylphosphatidylethanolamine; LPI, lysophosphatidylinositol; CL, cardiolipin; PE, phosphatidylethanolamine; LPE, lysophosphatidylethanolamine; PI, phosphatidylinositol; PC, phosphatidylcholine; LPA, lysophosphatidic acids; dMePE, dimethylphosphatidylethanolamine; PS, phosphatidylserine; phSM, phosphatidylsphingomyelin; CerPE, ceramide phosphoethanolamine; SPH, sphingosine phosphate; LSM, lysosphingomyelin; Hex1Cer, monohexosylceramide; GM3, monosialodihexosylganglioside; SM, sphingomyelin; Hex2Cer, dihexosylceramide; Cer, ceramide Hex3Cer, trihexosylcermide; CmE, ceramide monohexoside; SiE, sphingosine ester; ZyE, zymosteryl; ChE, cholesterolester; StE, sterol esters; AcCa, acylcarnitine; AEA, anandamide. (**C**,**D**) Identify differential lipids between overweight (**C**) or obese (**D**) and normal group using three distinct statistical algorithms, including *t*-test, PLSDA, and OPLSDA. (**E**) Enriched lion terms of differential lipids shared in both comparisons of obese vs. normal and overweight vs. normal. (**F**) Enriched lion terms of the unique differential lipids in the comparison of obese vs. normal.

To explore lipid alteration with increasing BMI, we profiled lipidomes in the serum of postoperative craniopharyngioma patients with different BMIs. A total of 1119 lipids from three groups were identified and classified into 5 major categories and 34 subcategories (Figure 3A and Figure S2A). In comparisons across different groups, four major categories, including GL, GP, SP, and ST, exhibited differential expressions to varying degrees. In contrast, the statistical difference of FA was observed only in the overweight comparison group (Figure 3B). The detailed differential lipids among the three groups were included in Table S4. This outcome suggests that the extent of weight gain differentially affects lipidomic profiles in postoperative craniopharyngioma patients. In the overweight vs. normal comparison, a total of 300 lipids out of 1119 were identified as significantly different through the *t*-test (Figure 3C and Figure S2B, and Table S4). Similar to metabolome, the PLS-DA and OPLS-DA models revealed distinct clustering of participants between the overweight and normal groups (Figure S2C,D). Applying the criterion of VIP > 1, PLS-DA identified a total of 335 key lipids, while OPLS-DA identified 344 key lipids (Figure 3C and Table S4). Then, an overlapping analysis was performed for differential lipids obtained from the three different methodologies, resulting in 296 discriminative lipids, comprising 154 upregulated and 142 downregulated (Figure 3C and Table S4). In the comparison between the obese and normal groups, a *t*-test identified 401 differential lipids out of a total of 1119 lipids (Figure 3D and Figure S2E, and Table S5). Applying the VIP > 1 criterion in the PLS-DA and OPLS-DA models resulted in identifying 323 and 330 key lipids, respectively (Figure 3D and Figure S2F,G, and Table S5). Through the overlapping analysis of differential lipids obtained from the three methodologies, 299 key lipids were identified, including 183 upregulated and 116 downregulated (Figure 3D and Table S5). Unlike the metabolomic profile, the quantity of differential lipids does not increase with higher BMI in postoperative craniopharyngioma patients. Nevertheless, changes in lipid composition across different levels of weight gain were evident. This implies a marked divergence in the patterns of alterations among metabolites and lipids in postoperative craniopharyngioma patients with different BMIs.

### 3.5. Lipid Ontology Enrichment Analysis of Serum from Postoperative Craniopharyngioma Patients

For a comprehensive examination of the lipidomic data, the discriminative lipids were classified into those shared by both comparisons and those unique to obese vs. normal (Figure S2H). To clarify the function of these differential lipids, we conducted a lipid ontology (LION) analysis, which is an ontology correlating lipid species with biophysical, chemical, and biological information. The differential lipids commonly observed in both comparisons were enriched in 10 lipid ontology terms, with particular emphasis on the top three terms, including negative intrinsic curvature, diacylglycerophosphoglycerophosphodiradylglycerols, and headgroup with a negative charge (Figure 3E). These three terms were mainly related to the function of the membrane. This suggests that the alteration of plasma membrane function is involved in weight gain in postoperative craniopharyngioma patients. Compared to the overweight vs. normal weight comparison group, the unique differential lipids in the obese vs. normal weight comparison group were notably enriched in an additional 10 terms, with specific attention to three terms: C22:4, C18:0, and glycerophosphoinositols (Figure 3F). This suggests that the alteration of these three lipids contributed to a more significant increase in the BMI of postoperative craniopharyngioma patients.

### 3.6. Correlations between Altered Metabolites and Differential Clinical Characteristics

**Figure 4:**
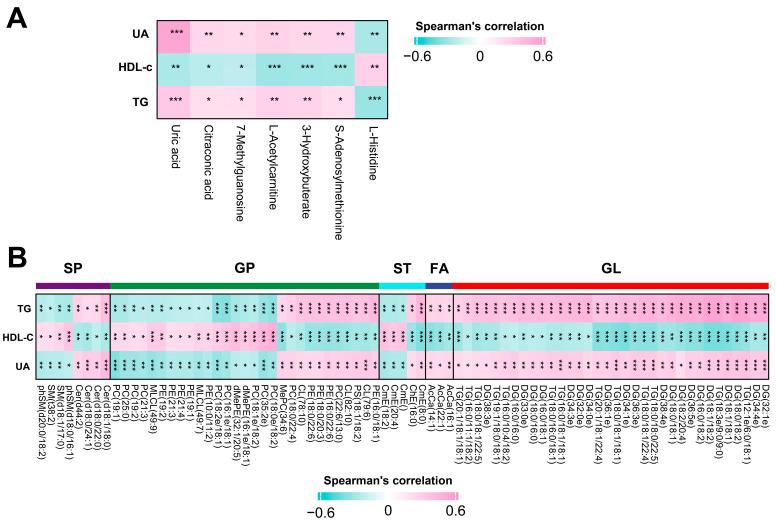
Correlations between altered metabolites and lipids and differential clinical characteristics. (**A**,**B**) Heatmap plot of correlation between differential clinical characteristics, including UA, TG, and HDL-C, and all differential metabolites (**A**) or lipids (**B**) upon overweight and obese group compared with normal group respectively. The color scale indicates the values of the Spearman correlation coefficient, with red representing higher values and green representing lower values. UA, uric acid; TG, triglyceride; HDL-C, HDL cholesterol. * *p* < 0.05, ** *p* < 0.01, *** *p* < 0.001.

In clinical characteristics analysis, we found that the levels of UA, HDL-c, and TG were distinctly different between the three groups. To explore the correlation between differential metabolites with these clinical characteristics, we performed a Spearman correlation analysis. A total of 8 metabolites, including UA, citraconic acid, 7-methylguanosine, l-acetylcarnitine, 3-hydroxybuterate, s-adenosylmethionine, and l-histidine, were found to be correlated with the levels of UA, HDL-C, and TG (Figure 4A and Table S6). Interestingly, the correlations observed between these differential metabolites and HDL-C displayed a pattern opposite to the observed trend for UA and TG. For instance, l-histidine and s-adenosylmethionine, intermediates of methylhistidine metabolism, exhibited opposite correlations with HDL-C, UA, and TG. This indicates an involvement of methylhistidine metabolism imbalance in postoperative metabolic disorders of craniopharyngioma patients. Another differential metabolite, UA, exhibited a positive correlation with UA as detected by a clinical biochemical analyzer, underscoring the high detection accuracy of the mass spectrometer. Concurrently, the metabolite UA is positively correlated with TG and negatively correlated with HDL-c. These findings align with the conclusion that UA contributes to lipid metabolism disorders.

Additionally, 3-Hydroxybutyrate, a participant in ketone body metabolism, is positively correlated with TG and UA and negatively correlated with HDL-c. This suggests that ketone body metabolism also mediates lipid and UA metabolism disorders in postoperative craniopharyngioma patients. Meanwhile, we also conducted a correlation analysis between altered metabolites and no differential clinical characteristics. We found that few altered metabolites were related to these no differential clinical characteristics (Table S7). It implies that these no differential clinical characteristics have no appreciable effect on the differential metabolites.

### 3.7. Correlations between Altered Lipids and Differential Clinical Characteristics

A Spearman correlation analysis was conducted to investigate the relationship between these differential lipids and differential clinical characteristics. A total of 79 lipids, including 5 major categories, were correlated with the levels of UA, HDL-C, and TG (Figure 4B and Table S6). Similar to metabolome, the correlations between these distinctive lipids and HDL-C exhibited a pattern opposite to the observed trend for UA and TG. Within these five major lipid classes, GP and GL predominantly constitute the majority, indicating their significant implication in postoperative craniopharyngioma patients’ lipid and UA metabolism disorders. For the GL lipid class, lipid ontology analysis revealed these lipids primarily participated in lipid storage and lipid droplets. The GP lipid class enrichment analysis suggests that these lipids are predominantly localized in the mitochondria, membrane component, and endoplasmic reticulum (Table S6). Notably, these enriched terms play a crucial role in metabolic regulation, indicating that GP and GL may participate in metabolic disorders by influencing lipid storage and the function of mitochondria, cell membranes, and endoplasmic reticulum.

Meanwhile, we also conducted a correlation analysis between altered lipids and no differential clinical characteristics. Unlike the metabolomics data, the differential lipids show a strong correlation with no differential clinical characteristics (Table S7). It implies that these no differential clinical characteristics significantly influence the differential lipids.

### 3.8. Screening of Key Metabolites and Lipids Associated with BMI

**Figure 5:**
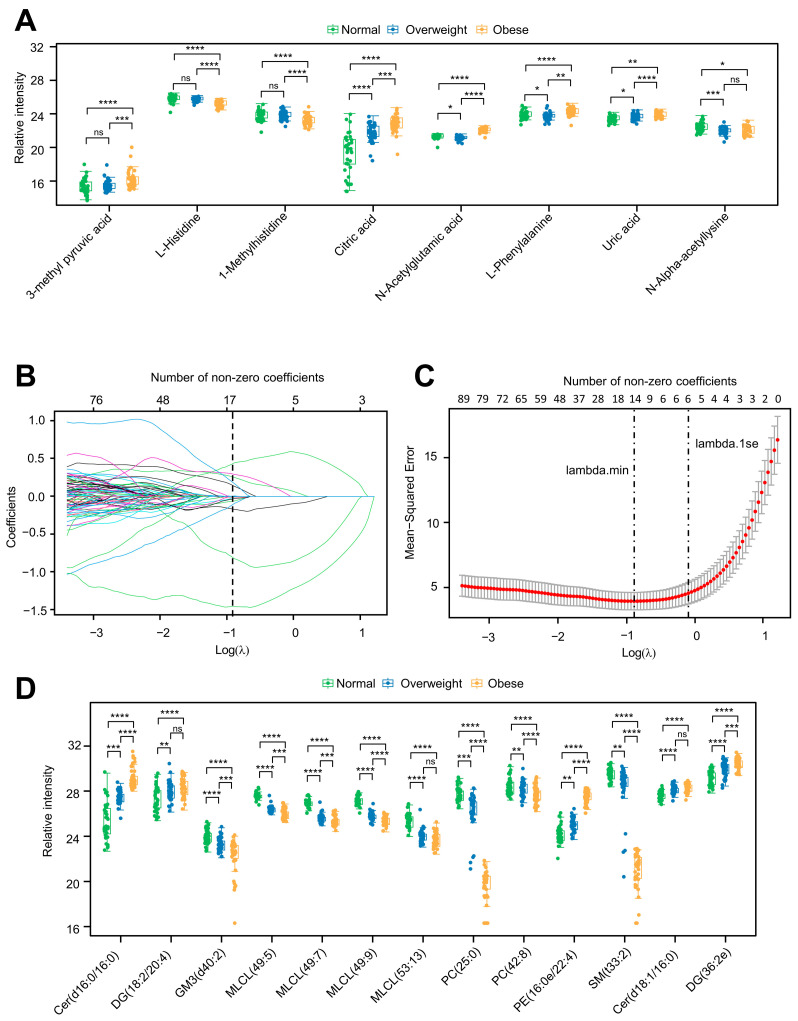
Screening of key metabolites and lipids related to BMI based on liner or LASSO regression. (**A**) Box plots of eight metabolites associated with BMI were selected by linear regression models. * adjust *p* < 0.05, ** adjust *p* < 0.01, *** adjust *p* < 0.001, **** adjust *p* < 0.0001, ns: not significant. (**B**) The variation characteristics of the coefficient of lipids related to BMI by LASSO model. (**C**) Cross-validation for tuning lipids selection in the LASSO regression model. Dotted vertical lines were drawn at the optimal values with Lambda (log) by using the minimum criteria and the one standard error of the minimum criteria (the 1st criteria). (**D**) Box plot of twelve lipids associated with BMI selected by LASSO regression model. ** adjust *p* < 0.01, *** adjust *p* < 0.001, **** adjust *p* < 0.0001, ns: not significant.

We conducted a stepwise linear regression analysis with BMI as the dependent variable and metabolites as independent variables to obtain the critical metabolites associated with BMI. Linear regression analysis identified eight key metabolites: four positively correlated with BMI and the remaining negatively correlated with BMI (Table 2). Then, we performed a further analysis using adjustments for age, gender, postoperative time to serum collection, HDL-c, UA, TG, drug treatment, and comorbidities. Interestingly, after correcting the potential confounding factors, the above eight metabolites still significantly correlate with BMI (Table 2). These data indicate that age, gender, postoperative time to serum collection, HDL-c, UA, TG, drug treatment, and comorbidities do not affect the metabolite profile. Multivariate linear regression revealed the level of 3-methyl pyruvic acid, citric acid, n-acetylglutamic acid, and UA, positively associated with BMI, shows a gradual increase with the increase in BMI (Figure 5A). Conversely, the level of 1-Methylhistidine, L-Histidine, L-Phenylalanine, and N-Alpha-acetyllysine, which are negatively correlated with BMI, gradually decreases with the increase in BMI (Figure 5A). Then, we conducted a validation of the linear model, and validation results suggest that the constructed model is successful (Figure S3A–F).

Due to the presence of multicollinearity among differential lipids (Figure S3G), we performed the following LASSO regression analysis adjusting for age, gender, postoperative time to serum collection, HDL-c, UA, TG, drug treatment, and comorbidities to obtain the key lipids associated with BMI (Figure 5B). LASSO regression analysis identified 13 key lipids, including Cer (d16:0/16:0), DG (18:2/20:4), GM3 (d40:2), MLCL (49:5), MLCL (49:7), MLCL (49:9), MLCL (53:13), PC (25:0), PC (42:8), PE (16:0e/22:4), SM (t33:2), Cer (d18:1/16:0), and DG (36:2e) (Figure 5C,D). Similar to the metabolome, among these 13 lipids, the levels of 5 lipids increase with the rise in BMI, while the levels of another 8 lipids decrease with the increase in BMI. These results indicate that there may be certain lipids and metabolites that can predict the occurrence of postoperative obesity in craniopharyngioma patients.

### 3.9. Receiver Operating Characteristic Curve Analysis

**Figure 6:**
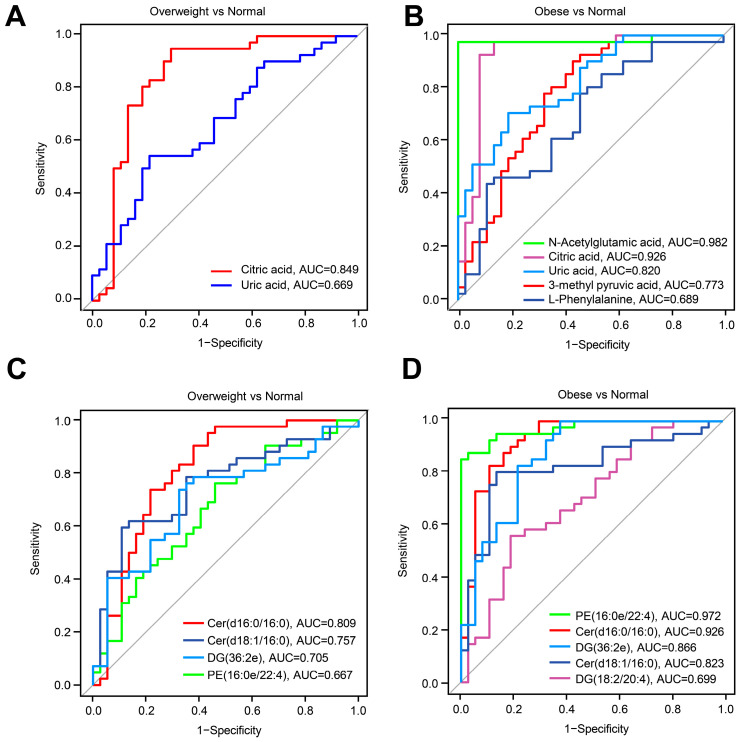
Receiver operating characteristic curve analysis. (**A**,**B**) ROC curve analysis for the predictive power of key metabolites selected by linear regression for distinguishing overweight group (**A**) or obese group (**B**) from normal weight group. (**C**,**D**) ROC curve analysis for the predictive power of key lipids selected by LASSO regression for distinguishing the overweight group (**C**) or obese group (**D**) from the normal weight group.

To further evaluate the usefulness of lipid and metabolite signatures for postoperative obesity in craniopharyngioma patients, we conducted ROC analysis adjusting for age, gender, postoperative time to serum collection, HDL-c, UA, TG, drug treatment, and comorbidities to identify key lipids and metabolites from the previous screening. ROC analyses comparing overweight and normal group subjects revealed that citric acid and UA exhibited AUC values of 0.849 and 0.669, respectively (Figure 6A). Subsequently, we analyzed the ROC curves for each metabolite between overweight and normal group subjects, showing that n-acetylglutamic acid, citric acid, UA, 3-methyl pyruvic acid, and l-phenylalanine had AUC values of 0.982, 0.926, 0.820, 0.773, and 0.689, respectively. These results suggest that citric acid and UA have the predictive potential for postoperative obesity and overweight in craniopharyngioma patients, while n-acetylglutamic acid, 3-methyl pyruvic acid, and l-phenylalanine precisely predict the occurrence of postoperative obesity in craniopharyngioma patients. In terms of lipids, ROC analysis between overweight and normal group subjects identified Cer (d16:0/16:0), Cer (d18:1/16:0), DG (36:2e), and PE (16:0e/22:4) as potential predictors for postoperative overweight in craniopharyngioma patients. ROC analysis between obese and normal group subjects identified PE (16:0e/22:4), Cer (d16:0/16:0), DG (36:2e), Cer (d18:1/16:0), and DG (18:2/20:4) as potential predictors for postoperative obesity in craniopharyngioma patients. These findings collectively suggest that metabolomics and lipidomics offer a promising tool for discriminating postoperative obesity in craniopharyngioma patients, and lipidomics exhibits higher sensitivity.

## 4. Discussion

Craniopharyngioma patients often experience a significant decline in their quality of life after surgery, with almost half developing HO [8]. This condition can lead to a high risk of mortality from cardiovascular disease. Unfortunately, there is currently no long-term effective treatment for this condition. So far, only studies with small sample sizes have examined clinical indicators of glucose and lipid metabolism in patients after craniopharyngioma surgery [26]. The impact of HO on glucose and lipid metabolism following craniopharyngioma surgery has not received much attention. Our research center has published articles indicating that approximately one-third of patients with craniopharyngioma become obese after surgery and are prone to abnormal glucose and lipid metabolism [5]. Therefore, it is necessary for us to comprehensively describe the metabolite and lipid profiles of patients after craniopharyngioma surgery using multi-omics techniques.

Metabolomic and lipidomic analysis in this study revealed significant differences in metabolites, lipids, and metabolic pathways among the three groups, which had rarely been reported in the past. Notably, metabolites including S-Adenosylmethionine, 3-Hydroxybuterate, L-Acetylcarnitine, 7-methylguanosine, Citraconic acid, and UA had a significant positive correlation with clinically measured serum TG and UA levels and a negative correlation with HDL-c levels. The correlation between L-Histidine and the clinical indicators mentioned above is opposite to what was expected. This study suggests that high levels of histidine in serum may be associated with improved body weight, blood lipids, and UA levels. This aligns with published meta-analyses indicating that dietary histidine supplementation can decrease the risk of central obesity and improve glucose metabolism outcomes [27].

Clinically, metabolic dysfunction-associated fatty liver disease (MAFLD) may develop after dysfunction of the hypothalamic-pituitary-target organ axis following neurosurgery, which can be treated with appropriate hormone replacement therapy [28]. Previous retrospective studies from our group have shown that postoperative patients with craniopharyngioma are prone to HO, hyperuricemia, and MAFLD [5]. This study aims to identify the metabolite biomarkers of HO. We analyzed the prevalence of pituitary function impairment and drug usage between groups, which showed no significant statistical differences between cases. However, there are still substantial differences in UA levels between groups. This indicates a close relationship between UA and HO, and it is worth exploring other potential mechanisms. Animal experiments have confirmed that UA can cause hypothalamic inflammation through the NF-κB signaling pathway [29]. A mediation link between obesity and UA with the development of MAFLD was observed in a clinical cohort study [30]. Based on the research findings, we speculate that UA may significantly contribute to the onset of obesity after craniopharyngioma surgery. Our team will investigate the occurrence of MAFLD and hyperuricemia in HO and explore their relationship and their precise mechanisms in future studies.

In a recent study, it was found that specific components of SP (Sphingomyelin), GP (Glycerophospholipid), and ST (Steroid) were negatively correlated with UA, which had a positive effect on improving lipid metabolism. On the other hand, FA (fatty acid) and GL (glycerolipid) showed opposite effects on blood lipids and UA. Previously, our group had identified three phosphatidylcholine (PC) lipid species, PC (14:0/18:3), PC (31:1), and PC (32:2), that were significantly associated with weight change in three different weight-loss intervention cohorts [31]. The previous study of the sellar region disease diagnosis and treatment center of the investigator has also investigated the lipid metabolite profile of individuals with pituitary tumors that produce growth hormones. It’s found that PE (22:6/16:0) and LysoPC (16:0) correlated with cardiac structure and function in acromegaly patients, which may contribute to the risk of cardiovascular complications [32]. More studies are needed to verify the relationship between lipid metabolites and metabolic diseases and their mechanisms. In Pikó’s study [33], seven key lipids, including PE P-16:0/20:3, TG 20:4_33:1, TG 22:6_36:4, TG 18:3_33:0, Hex-Cer 18:1;O2/22:0, LPC 18:2, and PC 18:1_18:1 showed a strong significant association with BMI, while our study found 13 lipids associated with BMI. However, the results of the two studies are completely different. This discrepancy may be due to differences in the ethnicities of the study populations and the varying health conditions of the subjects.

This study has some limitations, primarily its single-center cross-sectional design. For instance, it does not include a control group before and after craniopharyngioma surgery and cannot represent all geographical and ethnic characteristics. We plan to conduct a multicenter, pre-, and post-operative self-control study and a longitudinal cohort study with multiple follow-up time points. Additionally, although the majority of the measured metabolites do not exhibit significant changes after undergoing multiple freeze-thaw cycles [34], the action of serum freeze-thawing can reduce signals in sensitive metabolites. This signal reduction can limit the detection of metabolites that could assist in interpreting involved networks, especially redox-sensitive metabolites [35]. We also plan to optimize the conditions under which blood samples are collected and processed and to manage multiple body fluid samples, such as urine, in future studies to examine the effect of UA excretion capacity on metabolism. Additionally, dietary habits and psychological status could indeed have a significant impact on the metabolic disorders of postoperative obesity among adult-onset craniopharyngioma, which were lacking in this research. This information should be collected in future research.

This study found no significant differences in gender across the groups after stratifying by BMI. Nonetheless, sex differences in metabolic disorders are an area that warrants thorough investigation. In future investigations, conducting a gender-stratified analysis would be ideal if we can obtain a sufficiently large sample size. Moreover, it is essential to investigate further the effects and mechanisms of the differential metabolites that were found to impact body weight significantly in this study. This can be achieved through additional experiments, such as functional verification using cell or animal models and genetic and pharmacological methods to understand the underlying mechanisms of gain and loss of function. Finally, it is hoped that isotope tracing technology will be used in some patients to provide sufficient evidence for the mechanistic study of metabolic pathways. Our ultimate goal is to successfully prevent or treat hypothalamic obesity in patients after craniopharyngioma surgery. Further studies on the metabolic and lipid profile changes before and after weight loss in this population will help us better understand the underlying mechanisms and identify potential therapeutic targets.

## 5. Conclusions

In conclusion, we presented the serum metabolomic and lipidomic profile by the high-throughput tandem mass spectrometry, revealing the signature of postoperative obesity among adults with adult-onset craniopharyngioma. These differential metabolites and lipids, including n-acetylglutamic acid, citric acid, UA, 3-methyl pyruvic acid, l-phenylalanine, PE(16:0e/22:4), Cer(d16:0/16:0), DG (36:2e), Cer(d18:1/16:0), and DG(18:2/20:4) may be closely linked to HO and other metabolic disorders. In particular, citric acid and UA metabolites are closely related to weight control, lipid metabolism, and UA metabolism, highlighting their important roles in maintaining the body’s metabolic balance. It’s important to note that this is the first study to use metabolomics technology to examine postoperative patients with craniopharyngioma and their metabolism-related conditions. This enhances our understanding of these conditions both domestically and internationally, broadens research perspectives on HO, and fills gaps in related fields. In addition, a comprehensive and methodical analysis of the molecular mechanisms in animal models is necessary. Furthermore, additional prospective clinical studies are required for validation.

## Figures and Tables

**Table 1:** Demographics and characteristics of study participants.

Characteristic	Normal (*n* = 37)	Overweight (*n* = 42)	Obese (*n* = 41)	*p*-Value ^a^
Postoperative duration (months)	6.50 [3.30, 13.10]	7.65 [3.42, 56.30]	5.60 [3.60, 12.20]	0.422
Age (years)	36.00 [29.00, 58.00]	44.00 [31.25, 56.00]	40.00 [28.00, 53.00]	0.702
Male (%)	18 (49%)	28 (67%)	26 (63%)	0.227
Weight (kg)	59.93 ± 6.10	74.04 ± 9.45 ***	86.64 ± 11.13 ***^,†††^	<0.001
Height (cm)	164.32 ± 7.01	168.29 ± 8.87	166.80 ± 9.19	0.117
BMI (Kg/m^2^)	22.68 [21.26, 23.36]	25.97 [24.95, 27.07] ***	30.46 [29.07, 32.31] ***^,†††^	<0.001
SBP (mmHg)	117.70 ± 14.65	120.24 ± 16.30	118.66 ± 12.49	0.736
DBP (mmHg)	74.00 [70.00, 79.00]	79.00 [70.00, 86.75]	78.00 [72.00, 84.00]	0.070
FPG (mmol/L)	4.80 [4.40, 5.10]	5.00 [4.60, 5.47]	5.00 [4.50, 5.70]	0. 097
HbA1c (%)	5.55 [5.30, 5.82]	5.75 [5.40, 6.07]	5.90 [5.40, 6.70]	0.055
TC (mmol/L)	4.59 [4.26, 5.87]	4.86 [4.45, 6.09]	5.27 [4.21, 5.69]	0.666
TG (mmol/L)	1.57 [1.22, 2.30]	2.31 [1.59, 3.20] *	2.6 [1.81, 3.64] **	0.006
HDL-c (mmol/L)	1.13 [0.89, 1.44]	0.98 [0.82, 1.11]	0.91 [0.73, 1.08]	0.019
LDL-c (mmol/L)	2.72 [2.28, 3.47]	2.81 [2.25, 3.53]	3.11 [2.36, 3.81]	0.670
UA (mol/L)	0.34 ± 0.10	0.40 ± 0.09	0.44 ± 0.13 ***	<0.001
Hypertension (%)	2 (5%)	8 (19%)	7 (17%)	0.179
Diabetes (%)	3 (8%)	7 (17%)	13 (32%) *	0.027
Hyperlipidemia (%)	22 (59%)	34 (81%) *	36 (88%) *	0.009
Hyperuricemia (%)	12 (32%)	18 (43%)	26 (63%) *	0.020
Hypopituitarism (%)	35 (95%)	41 (100%)	42 (100%)	0.102

SBP, systolic blood pressure; DBP, diastolic blood pressure; BMI, body mass index; FPG, fasting plasma glucose; HbA1c, glycated hemoglobin; TC, total cholesterol; Triglyceride, TG; HDL-c, high-density lipoprotein cholesterol; LDL-c, low-density lipoprotein cholesterol; UA, uric acid. *p*-value ^a^ refer to comparison among three group; * *p* < 0.05, ** *p* < 0.01, *** *p* < 0.001 vs. normal group with Bonferroni correction; ^†††^ *p* < 0.001 vs. overweight group with Bonferroni correction.

**Table 2:** Screening of key metabolites associated with BMI based on linear regression.

Term	Before Adjust		After Adjust ^a^	
	Estimate	*p*-Value	Estimate	*p*-Value
Intercept	26.56	<0.001	27.13	<0.001
1-Methylhistidine	−1.03	<0.001	−0.93	<0.001
3-methyl pyruvic acid	1.06	<0.001	1.14	<0.001
Citric acid	1.02	<0.001	1.04	<0.001
L-Histidine	−1.04	<0.001	−1.03	<0.001
N-Acetylglutamic acid	0.91	0.001	0.83	0.006
L-Phenylalanine	−0.66	0.02	−0.70	0.019
Uric acid	0.89	0.002	0.90	0.008
N-Alpha-acetyllysine	−0.52	0.026	−0.48	0.047

^a^ Age, gender, postoperative time for serum collection, HDL-c, UA, TG, drug treatment, and comorbidities were adjusted for analysis.

## Data Availability

The data presented in this study are available on request from the corresponding author. The data are not publicly available due to ethical reasons.

## Supplementary Materials

The following supporting information can be downloaded at: https://www.mdpi.com/article/10.3390/metabo14060338/s1, Figure S1: Identification of key metabolites in postoperative CP patients with increasing BMI; Figure S2: Identification of critical lipids in postoperative CP patients with increasing BMI; Figure S3: Model validation of metabolites and lipids; Table S1: Original clinical data and statistical analysis of clinical variables; Table S2: Statistical for overweight vs. normal on metabolic data; Table S3: Statistical for obese vs. normal on metabolic data; Table S4: Statistical for overweight vs. normal on lipidomic data; Table S5: Statistical for obese vs. normal on lipidomic data; Table S6: Correlation analysis of altered metabolites/lipids and differential clinical characteristics; Table S7: Correlation analysis of altered metabolites/lipids and other clinical characteristics.
