# Supplementary material for: Serum Metabolomic and Lipidomic Profiling Reveals the Signature for Postoperative Obesity among Adult-Onset Craniopharyngioma
Source: Metabolites. 2024 Jun 17;14(6):338. doi: 10.3390/metabo14060338 (PMC11205291; doi:10.3390/metabo14060338)
Supplement: Supplementary file 1 [file metabolites-14-00338-s001.zip › Supplemental file.docx]

**Supplemental Figure and Figure legends**


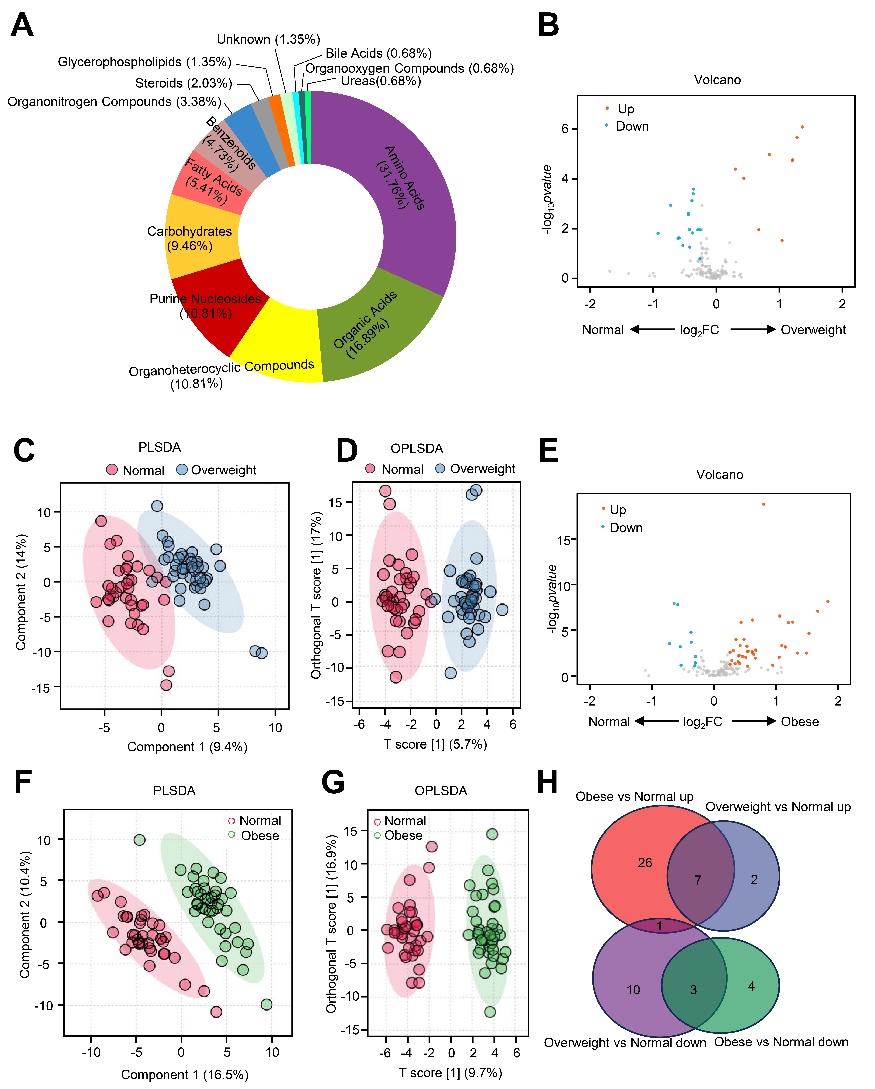


**Figure S1.** Identification of key metabolites in postoperative CP patients with increasing BMI. (A) Pie charts illustrating the proportion of each metabolite superclass. (B) Volcano plot of differential metabolites in overweight and normal groups with the algorithms of t-test. Each dot indicates a metabolite. (C-D) PLSDA (C) and OPLSDA (D) analysis plots of the samples in the overweight and normal groups. Each dot indicates a sample. (E) Volcano plot of differential metabolites in obese and normal groups with the algorithms of t-test. Each dot indicates a metabolite. (F-G) PLSDA (F) and OPLSDA (G) analysis plots of the samples in the obese and normal groups. Each dot indicates a sample. (H) Venn plot of shared and distinct differential metabolites upon overweight and obese group compared with normal group respectively.


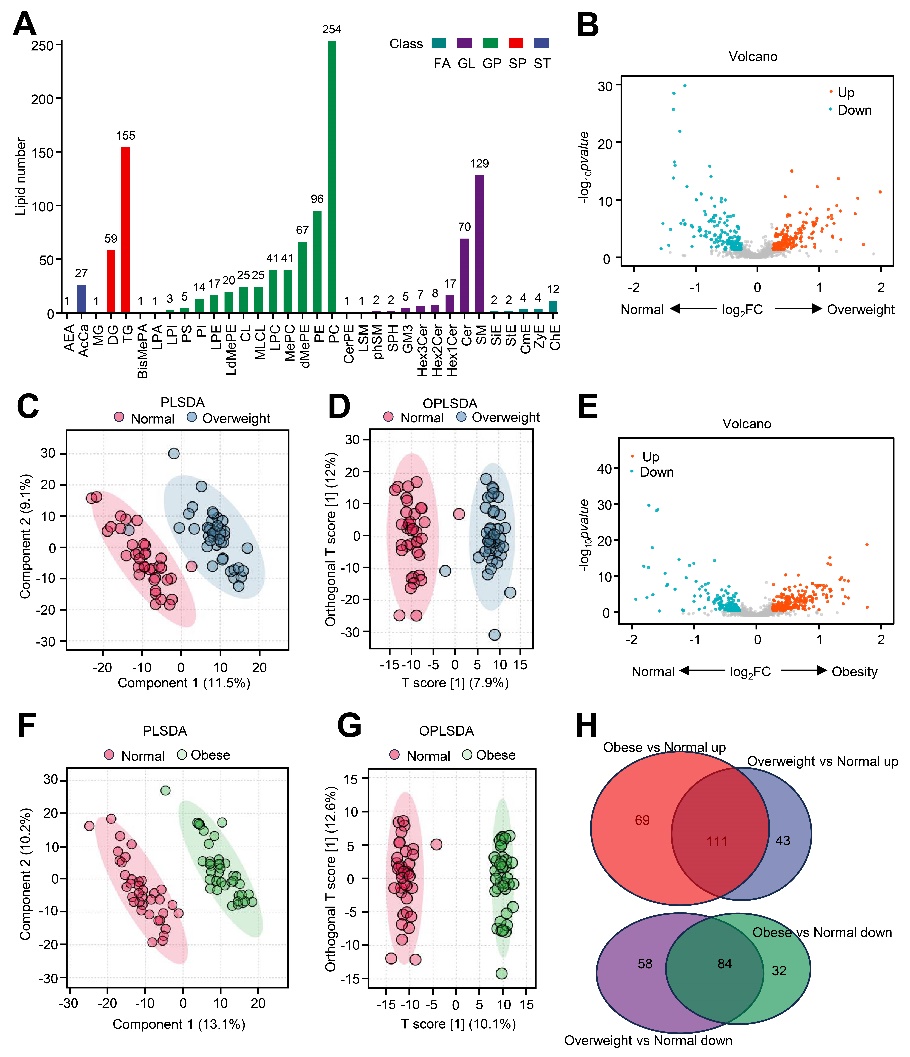


**Figure S2.** Identification of critical lipids in postoperative CP patients with increasing BMI. (A) Bar plots illustrating the number of each lipid superclass. (B) Volcano plot of differential lipids in the overweight and normal group with the algorithms of t-test. Each dot indicates a metabolite. (C-D) PLSDA (C) and OPLSDA (D) analysis plots of the samples in the overweight and normal groups. Each dot indicates a sample. (E) Volcano plot of differential lipids in obese and normal groups with the algorithms of t-test. Each dot indicates a lipid. (F-G) PLSDA (F) and OPLSDA (G)analysis plots of the samples in the obese and normal groups. Each dot indicates a sample. (H) Venn plot of shared and distinct differential lipids upon overweight and obese group compared with normal group respectively.


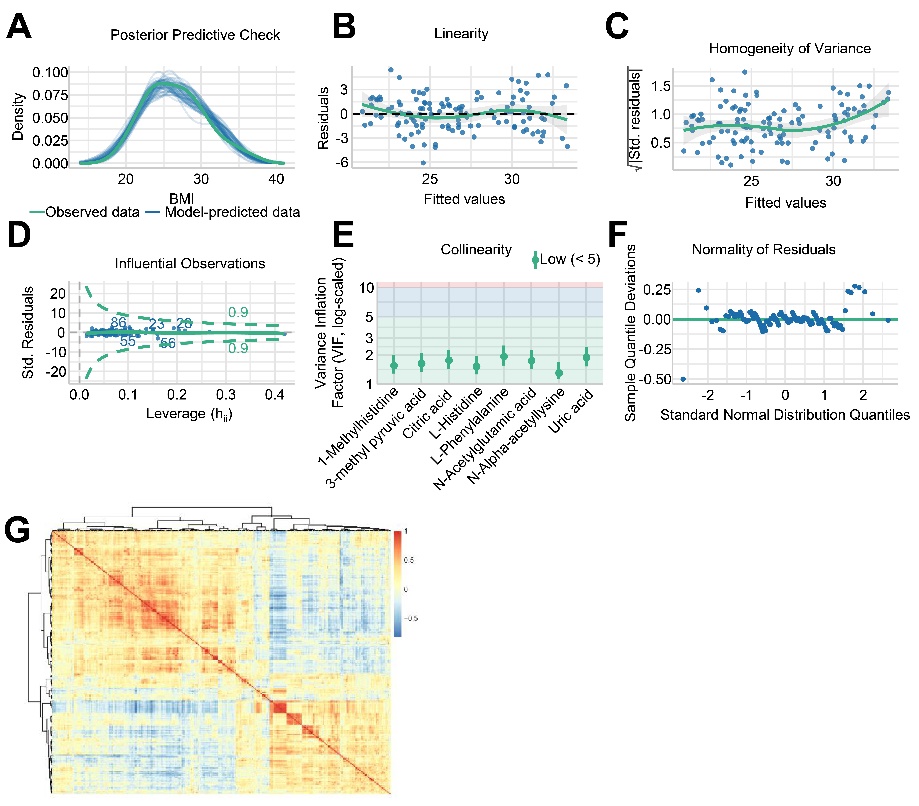


**Figure S3.** Figure S3. Model validation of metabolites and lipids. (A) Density plot of posterior predictive checks (B) Dot plot depicting the residuals of linearity assumption (C) Dot plot illustrating the homogeneity of variance. (D) Dot plot showing influential variable observations. (E) Bar plot depicting the collinearity of selected metabolites. (F) Dot plot illustrating the normality of residuals. (F) Heatmap plot of Spearman correlation between differential lipids. Each row and column represents a lipid. The color scale indicates the correlation coefficient values, with red representing higher values and blue representing lower values.
